# Regulation of epithelial-mesenchymal transition and metastasis by TGF-β, P-bodies, and autophagy

**DOI:** 10.18632/oncotarget.21871

**Published:** 2017-10-17

**Authors:** Shana D. Hardy, Aparna Shinde, Wen-Horng Wang, Michael K. Wendt, Robert L. Geahlen

**Affiliations:** ^1^ Department of Medicinal Chemistry and Molecular Pharmacology, and the Purdue Center for Cancer Research, Purdue University, West Lafayette, IN, 47907, USA

**Keywords:** P-body, autophagy, transforming growth factor beta (TGF-β), epithelial-mesenchymal transition (EMT), metastasis

## Abstract

Processing bodies (P-bodies) are ribonucleoprotein complexes involved in post-transcriptional mRNA metabolism that accumulate in cells exposed to various stress stimuli. The treatment of mammary epithelial cells with transforming growth factor-beta (TGF-β), triggers epithelial-mesenchymal transition (EMT), and induces the formation of P-bodies. Ectopic expression of the transcription factor TWIST, which stimulates EMT downstream of the TGF-β receptor, also promotes P-body formation. Removal of TGF-β from treated cells results in the clearance of P-bodies by a process that is blocked by inhibitors of autophagy. Activators of autophagy enhance P-body clearance and block EMT. Blockage of P-body formation by disruption of the gene for DDX6, a protein essential for P-body assembly, blocks EMT and prevents tumor cell metastasis *in vivo*. These studies suggest critical roles for P-body formation and autophagy in transitions of cancer cells between epithelial and mesenchymal phenotypes and help explain how autophagy functions to promote or suppress tumor cell growth during different stages of tumorigenesis.

## INTRODUCTION

The development of distant metastases is a multistep process wherein tumor cells undergo an epithelial-mesenchymal transition (EMT) to detach from the primary tumor mass, intravasate into blood or lymphatic vessels, and extravasate into ectopic sites [1, 2]. The EMT process can be induced by exposing epithelial cells to certain extracellular stimuli, most notably the growth factor TGF-β. By binding to its cognate receptors, TGF-β activates the SMAD pathway and other signaling pathways, leading to the expression of a network of transcription factors including TWIST, SNAI1/SNAIL, SNAI2/SLUG and ZEB1/2. When expressed on their own, each are capable of inducing EMT [3, 4]. Induction of EMT by TGF-β or the expression of transcription factors like TWIST or SNAI1 results in the altered expression of a common set of genes that comprise an EMT gene expression signature [4]. Specific miRNAs such as mir-200, which is downregulated during EMT, and mir-155, which is upregulated, are important modulators of EMT [5]. Consequently, a subset of alterations in mRNA levels during EMT are likely to be regulated post-transcriptionally.

miRNAs function as components of the RNA-induced silencing complex (RISC), which localizes to ribonucleoprotein complexes known as processing bodies or P-bodies [6, 7]. P-bodies house many proteins important for mRNA translational repression and decay and can assemble to large sizes in cells, particularly in response to external stress [8, 9] (although it is not certain that the formation of large complexes is always required for their activities [10, 11]). P-bodies also contain some proteins more classically associated with mRNA stability and mRNAs can exit P-bodies and cycle to polysomes for translation [8, 12]. In this regard, P-bodies are similar to stress granules, ribonucleoprotein particles that also form under stress conditions [13]. Stress granules share some components with P-bodies, but uniquely contain mRNAs within stalled translation initiation complexes and are thought to be important for stabilizing mRNAs in cells under stress.

Genetic analyses in yeast indicate that both P-bodies and stress granules are targeted for removal through macroautophagy (autophagy) [14]. Autophagy is best known as a mechanism to remove damaged organelles and misfolded proteins from normal cells and to provide nutrients and metabolites to stressed cells [15]. During autophagy, protein aggregates and organelles are surrounded by a double-membraned vesicle (autophagosome) that then fuses to a lysosome resulting in degradation of the encapsulated material. Autophagy has been associated with both oncogenic and tumor suppressive roles in cancer cells depending on the stage of tumorigenesis [16–18]. At early stages of tumorigenesis, autophagy is tumor suppressive such that negative modulators of autophagy promote tumor formation.

In this study, we describe a role for P-bodies and their removal through autophagy in EMT. While few P-bodies are present basally in cultured breast epithelial cells, these accumulate dramatically when cells are induced to undergo EMT in response to TGF-β or to the ectopic expression of TWIST. These P-bodies are cleared from cells following the removal of TGF-β by a process that is blocked by inhibitors of autophagy. The formation and persistence of P-bodies is required for EMT, which is blocked by promoters of autophagy or by the inhibition of P-body formation by the downregulation or deletion of DDX6, a protein required for P-body assembly. Consistent with this observation, the knockout of DDX6 blocks the *in vivo* formation of distant pulmonary metastases from orthotopic mammary tumors. These observations indicate a critical role for P-body formation in EMT and provide a mechanism by which autophagy can modulate tumorigenesis through P-body removal.

## RESULTS

### P-bodies are induced in mammary epithelial cells via canonical TGF-β signaling pathways

**Figure 1:**
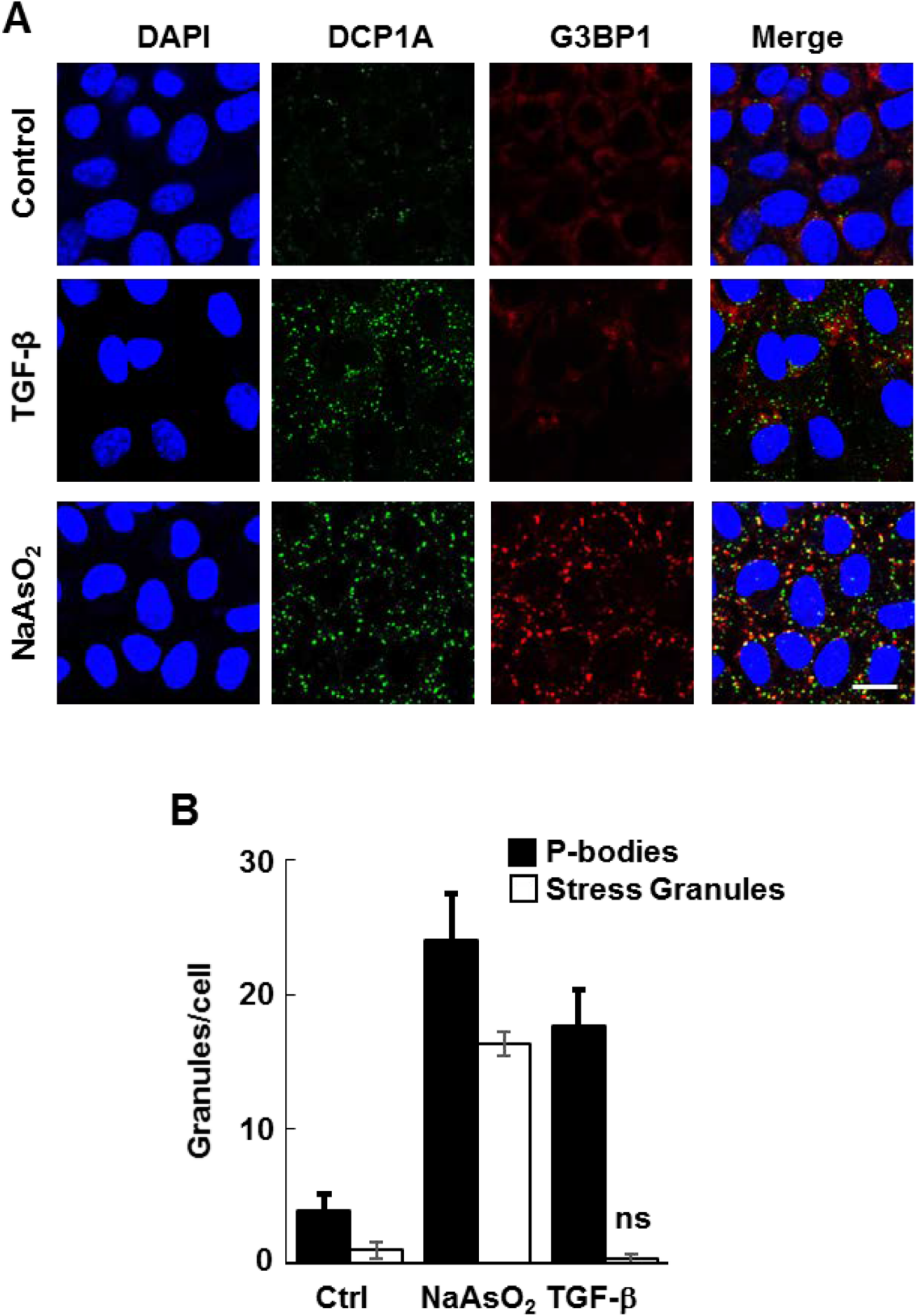
TGF-β induces the formation of P-bodies (**A**) NMuMG cells were treated without (Control) or with TGF-β for 24 h or sodium arsenite for 1 h, fixed and stained with anti-DCP1A (green) or anti-G3BP (red) to detect P-bodies or stress granules, respectively. Nuclei were stained with DAPI (blue). Bar, 10 μm. (**B**) The number of granules (P-bodies or stress granules) per cell was quantified (n > 100 cells per treatment). Data represents means ± SEM for triplicate experiments.

It was reported that the treatment of intestinal epithelial cells with TGF-β enhances the formation of P-bodies [19]. We asked if TGF-β also might stimulate P-body formation in breast epithelial cells. We tested this first in normal murine mammary gland (NMuMG) cells, which are well known to undergo EMT in response to TGF-β [20]. Treatment with TGF-β led to the induction of numerous P-bodies as shown by immunostaining with antibodies against DCP1A (Figure 1). In contrast, TGF-β did not significantly change the number of stress granules as detected using antibodies against G3BP1, a stress granule marker. P-bodies and stress granules both accumulate in cells in response to various external stresses including exposure to sodium arsenite [12]. In fact, NMuMG cells responded normally to sodium arsenite by forming both P-bodies and stress granules (Figure 1). Thus, TGF-β is a selective inducer of P-body accumulation.

**Figure 2:**
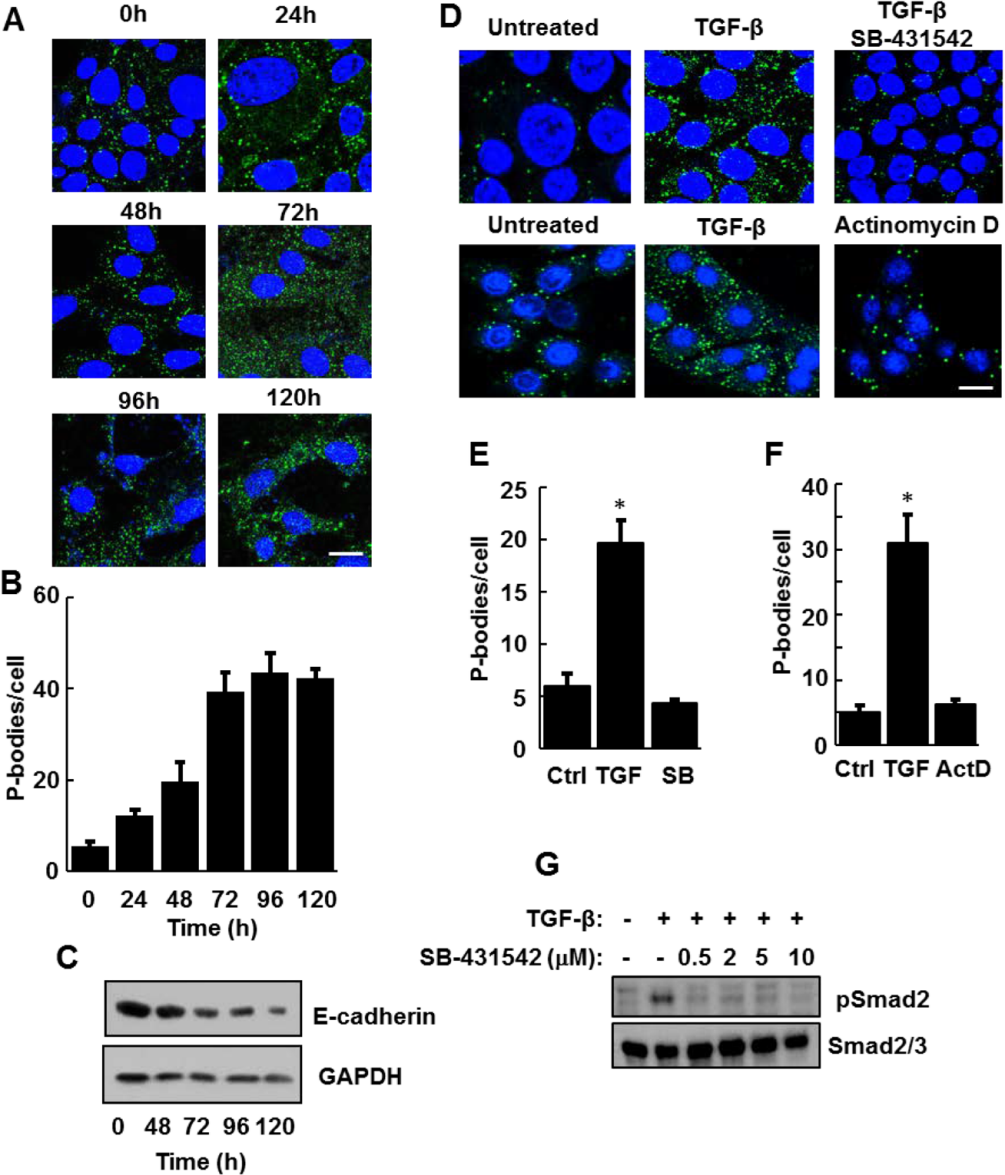
TGF-β receptor signaling is required for the induction of P-bodies (**A**) NMuMG cells were treated with TGF-β for the indicated times, fixed and stained with anti-DCP1A to visualize P bodies (green). Nuclei were stained with DAPI (blue). Bar, 10 μm. (**B**) The number of P-bodies/cell was quantified. Data represent average number of P-bodies per cell (n > 100 cells per treatment) ± SEM for triplicate experiments. (**C**) NMuMG cells were treated for the indicated times with TGF-β. Cell lysates were examined by Western blotting using antibodies against E-cadherin or glyceraldehyde 3-phosphate dehydrogenase (GAPDH). (**D**) NMuMG cells were not treated (Untreated), treated with TGF-β alone or with a combination of TGF-β and SB-431542 or TGF-β and Actinomycin D (ActD) for 24 h. Cells were fixed and stained with anti-DCP1A to visualize P bodies (green). Nuclei were stained with DAPI (blue). Bar, 10 μm. (**E** and **F**) Average number of P-bodies per cell ± SEM (*n* > 150 cells per treatment) from panel D were quantified from triplicate experiments. Data were analyzed by ANOVA, ^*^P < 0.001. (**G**) Lysates from NMuMG cells treated for 24 h without (-) or with (+) TGF-β in the absence (-) or presence (+) of the indicated concentrations of SB-431542 were examined by Western blotting using antibodies against phospho-SMAD2 (pSmad2) or Smad2/3.

To explore the nature of this response, we treated NMuMG cells with TGF-β over the course of 120 h and monitored P-body formation. Treatment with TGF-β resulted in a progressive increase in P-body numbers that reached a maximum around 72 h and persisted throughout the 5-days of treatment (Figure 2A and 2B). During this time, cells underwent a morphological change from an epithelial to a mesenchymal phenotype and exhibited a decrease in the expression of E-cadherin as expected of cells undergoing EMT (Figure 2C). Activation of the SMAD pathway is a major downstream mechanism by which TGF-β induces the expression of transcription factors such as SNAI1 and TWIST to drive EMT. Accordingly, treatment of NMuMG cells with the type 1 TGF-β receptor kinase inhibitor SB-431542 blocked TGF-β-induced P-body formation at a concentration that effectively prevented SMAD2 phosphorylation (Figure 2D, 2E and 2G). To determine if TGF-β-induced gene transcription was required for P-body formation, we treated cells with TGF-β in the presence or absence of the transcriptional inhibitor actinomycin D. Actinomycin D also blocked the TGF-β-mediated induction of P-bodies (Figure 2D and 2F).

**Figure 3:**
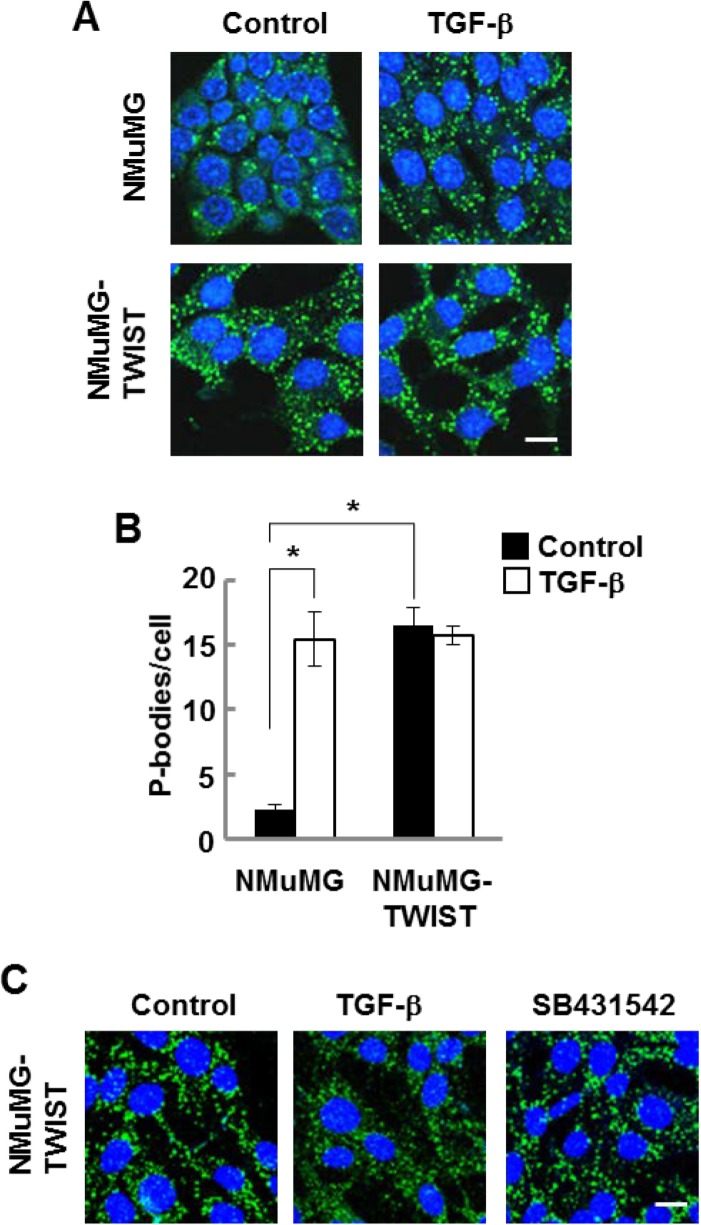
NMuMG cells expressing TWIST have high levels of P-bodies (**A**) NMuMG cells lacking or expressing TWIST were treated without (Control) or with TGF-β (10 ng/ml) for 24 h, fixed and stained with anti-DCP1A to visualize P bodies (green). Nuclei were stained with DAPI (blue). (**B**) Average number of P-bodies per cell ± SEM (*n* > 150 cells per treatment) was quantified from triplicate experiments. ^*^P < 0.001. (**C**) NMuMG cells expressing TWIST were treated without (Control) or with TGF-β or SB-431542 for 24 h, fixed and stained with anti-DCP1A to visualize P bodies (green). Nuclei were stained with DAPI (blue). Bar, 10 μm.

An important downstream effector of TGF-β signaling is the transcription factor, TWIST, whose expression is upregulated in response to the growth factor. The ectopic expression of TWIST alone induces EMT in breast epithelial cells [4]. Consequently, we examined NMuMG cells constructed to constitutively overexpress TWIST for the presence of P-bodies. TWIST-expressing cells, which had a mesenchymal phenotype as expected, contained abundant P-bodies (Figure 3A). The numerous P-bodies observed in these cells in the absence of TGF-β were not elevated further by addition of the growth factor (Figure 3A and 3B). Since the expression of TWIST in cells also leads to increased production of TGF-β [21], we treated the TWIST-expressing NMuMG cells with SB-431542 and looked for changes in P-body numbers. However, this treatment failed to repress P-body formation, indicating that the enhanced formation of P-bodies resulting from TWIST expression likely occurred downstream of the transcription factor (Figure 3C).

**Figure 4:**
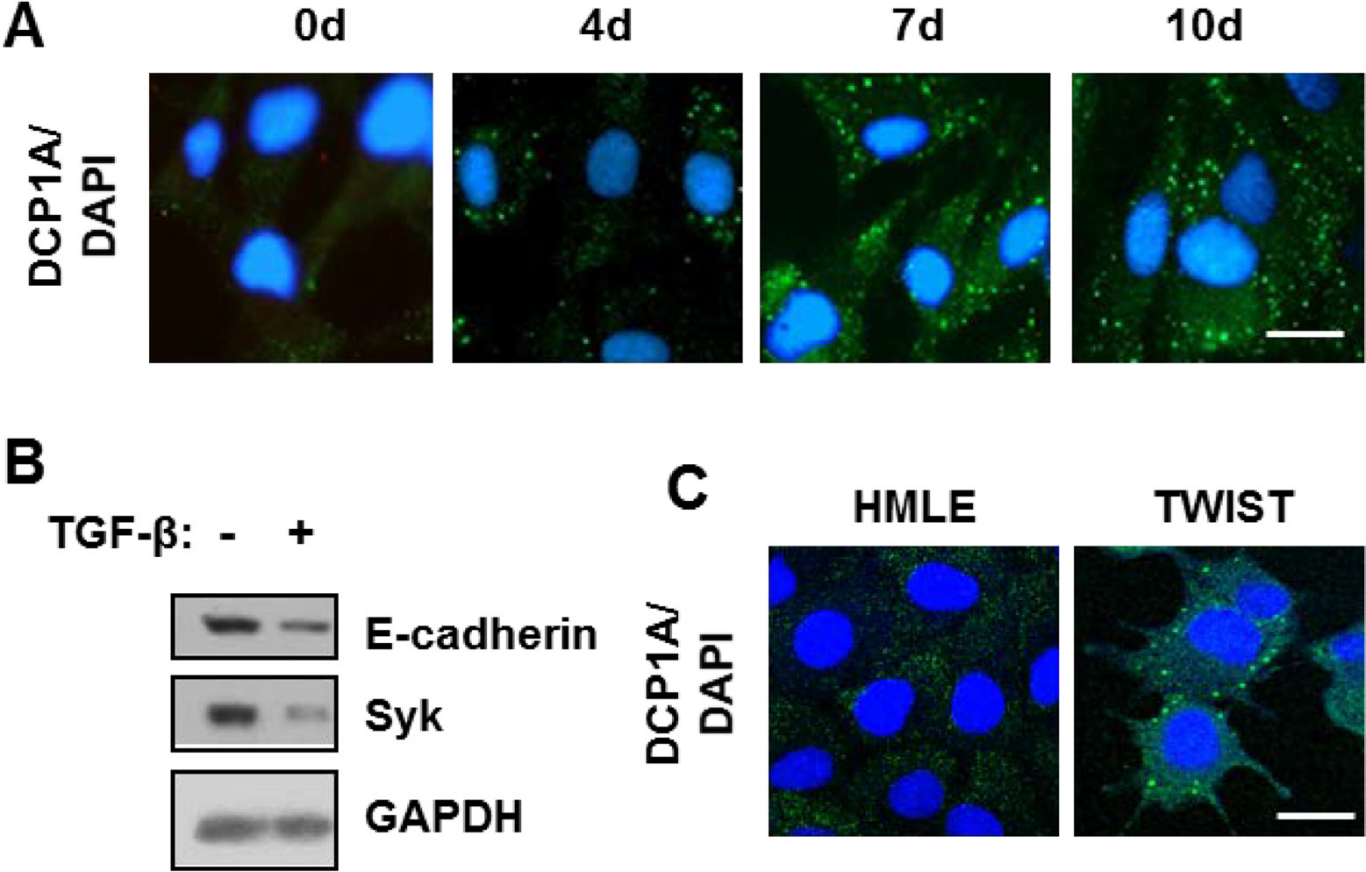
TGF-β promotes P-body accumulation HMLE and DG75 cells (**A**) HMLE cells were treated with TGF-β for the indicated times in days. Cells were fixed and stained with antibodies against DCP1A (green). Nuclei were stained with DAPI (blue). (**B**) HMLE cells were treated without (-) or with (+) TGF-β for 120 h. Cell lysates were examined by Western blotting using antibodies against E-cadherin, SYK, and GAPDH. (**C**) HMLE cells and HMLE cells expressing TWIST (TWIST) were fixed and stained with antibodies against DCP1A (green). Nuclei were stained with DAPI (blue). Bar, 10 μm.

To determine if the induction of P-bodies by TGF-β was restricted to NMuMG cells, we examined additional cell lines. Human mammary epithelial (HMLE) cells also undergo EMT in response to TGF-β, although this requires a more extended time of exposure to the growth factor as compared to NMuMG cells [22, 23]. Treatment of HMLE cells with TGF-β also induced the formation of P-bodies (Figure 4A). We confirmed the induction of EMT in treated cells using two representatives of the EMT gene expression signature: E-cadherin and the tyrosine kinase, SYK [4]. Treatment of HMLE cells with TGF-β resulted in the decreased expression of both markers (Figure 4B). The ectopic expression of TWIST in HMLE cells, like NMuMG cells, again resulted in cells containing a high constitutive level of P-bodies (Figure 4C).

### P-bodies are cleared from cells through autophagy

**Figure 5:**
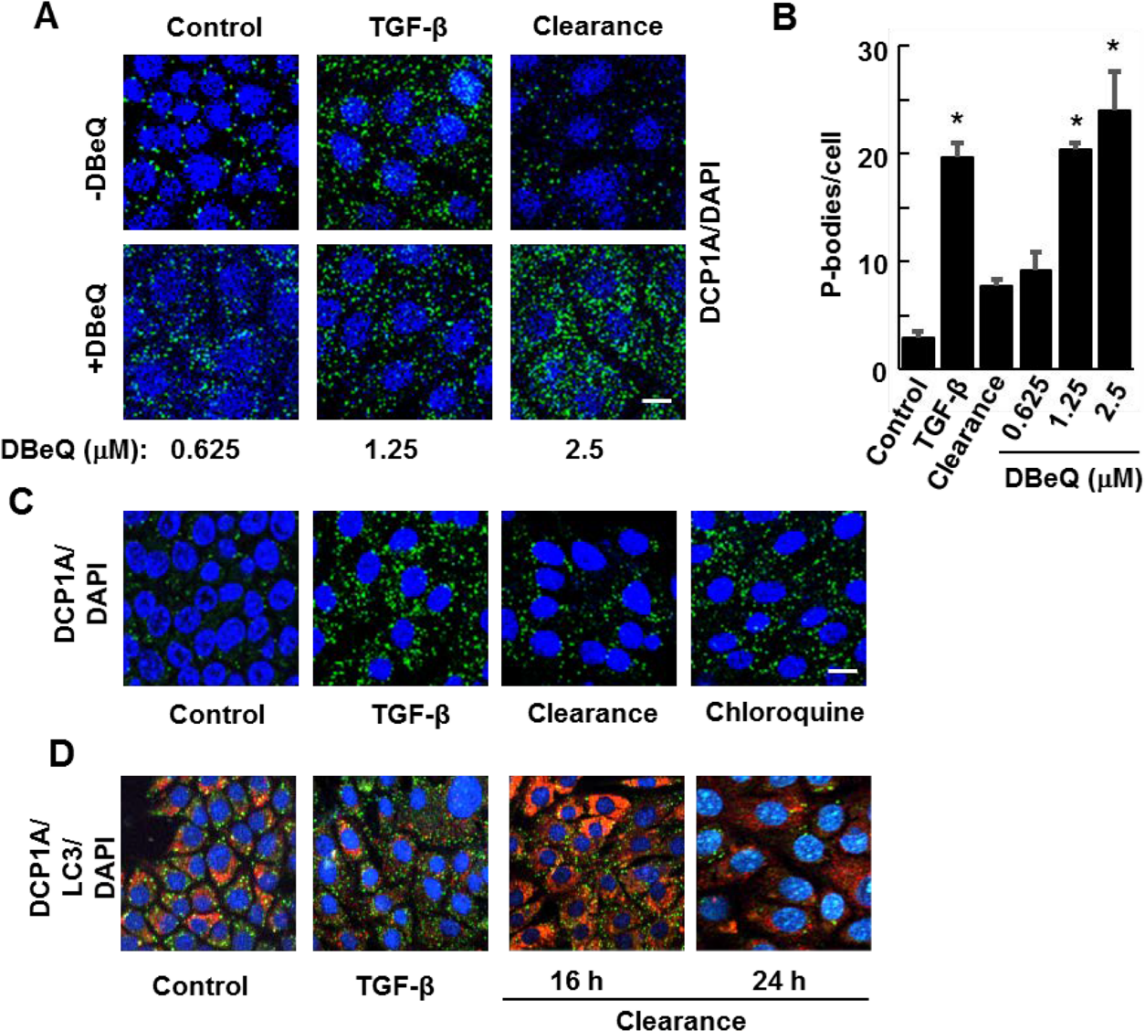
Inhibitors of autophagy block P-body clearance (**A**) NMuMG cells were treated without (Control) or with TGF-β for 24 h and then allowed to recover for 15 h (Clearance) in the presence of DMSO carrier or the autophagy inhibitor DBeQ at the concentrations indicated. Cells were fixed and stained using antibodies against DCP1A (green). Nuclei were stained with DAPI (blue). (**B**) Average number of P-bodies per cell ± SEM (*n* > 100 cells per treatment) was quantified from triplicate experiments. Data were analyzed by ANOVA, ^*^P < 0.001 (**C**) NMuMG cells were untreated (Control) or treated with TGF-β for 24 h. TGF-β was removed and cells were cultured in fresh media in the absence (Clearance) or presence of Chloroquine (10 µM) for 24 h. Cells were fixed and stained with anti-DCP1A to visualize P bodies (green). DAPI (blue) was used to visualize nuclei. Bar, 10 μm. (**D**) NMuMG cells were treated without (Control) or with TGF-β for 24 h and then allowed to recover for 16 or 24 h in the absence of TGF-β (Clearance). Cells were fixed and stained using antibodies against DCP1A (green) and LC3 (red). Nuclei were stained with DAPI (blue). Bar, 10 μm.

Genetic studies in yeast suggest a role for autophagy in the removal of both stress granules and P-bodies [14]. We showed previously that stress granules in mammary epithelial cells also are cleared through autophagy [24]. Consequently, we asked if autophagy was responsible for the clearance of P-bodies. To test this, we first induced P-body formation in NMuMG cells by treatment with TGF-β for 24 h. We then removed the stimulus and cultured cells in fresh media for 16 h in the absence or presence of increasing concentrations of the autophagy inhibitor DBeQ [25]. Cells were fixed and stained with antibodies against DCP1A to detect and count the number of P-bodies. P-bodies were readily cleared from cells following the removal of TGF-β, but clearance was blocked by the autophagy inhibitor and P-bodies continued to accumulate (Figure 5A and 5B). This strongly implied that autophagy was essential for clearing TGF-β-induced P-bodies. To confirm this, we monitored P-body clearance in the absence or presence of chloroquine, an inhibitor of autophagy that blocks endosome and lysosome fusion, a late stage process in autophagy. NMuMG cells were treated with TGF-β for 48 h and the growth factor was removed for 24 h in the presence or absence of the drug. The presence of chloroquine resulted in the retention of P-bodies (Figure 5C).

To examine further an association between autophagy and P-body clearance, we monitored the formation of autophagosomes by immunostaining for LC3, a protein that accumulates in these vesicles. Cells treated with TGF-β for 24 h contained only a low level of LC3-positive autophagosomes, a level lower than that of untreated cells. However, the subsequent removal of TGF-β for 16 h resulted in the robust accumulation of LC3-containing vesicles consistent with the formation of autophagosomes (Figure 5D). This elevated level of autophagosomes was transient, as many had disappeared by 24 h after removing TGF-β, a time at which most P-bodies have been cleared.

**Figure 6:**
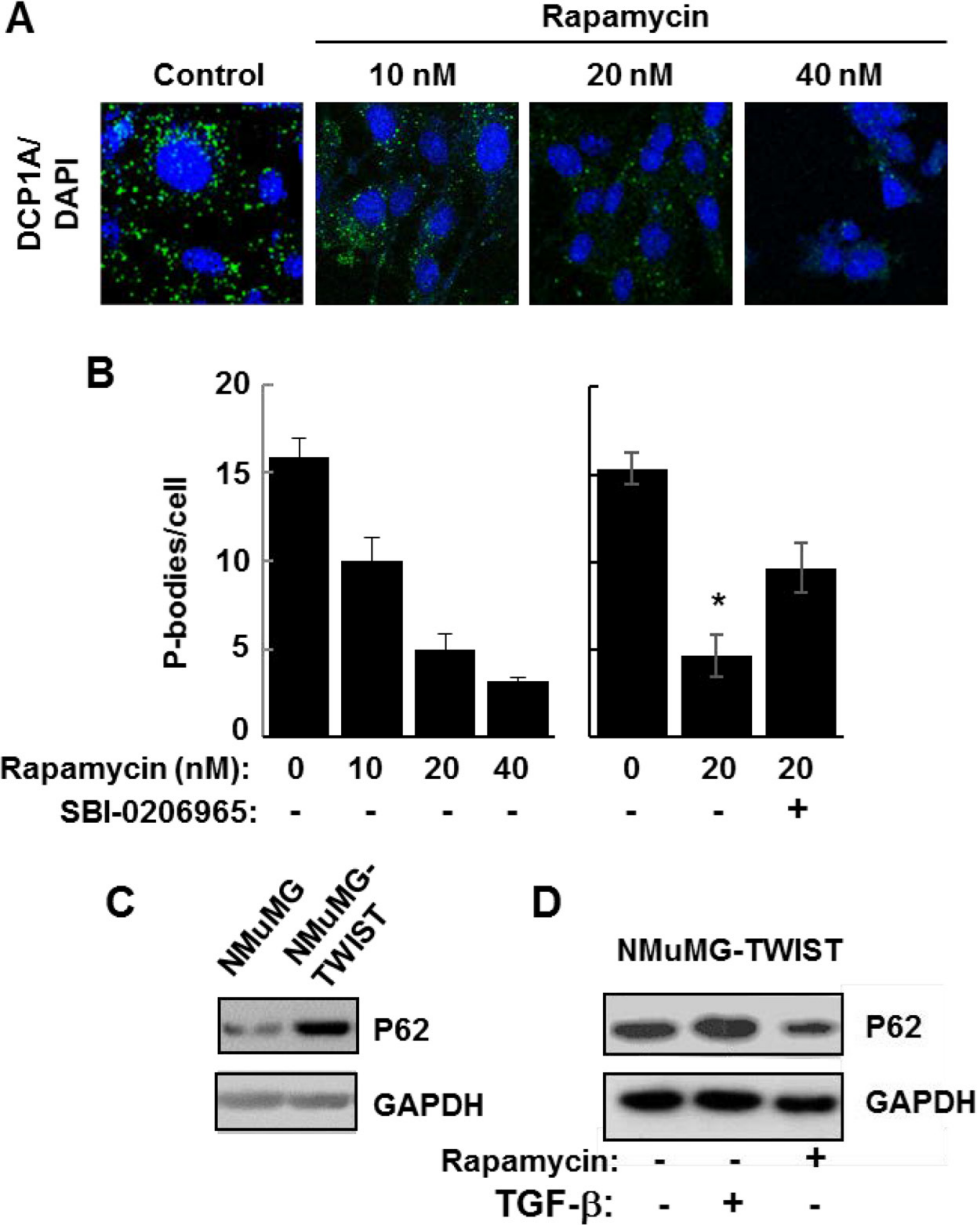
Autophagy promotes P-body clearance (**A**) NMuMG cells expressing TWIST were cultured in the absence (Control) or presence of rapamycin at the indicated concentrations for 24 h and stained with anti-DCP1A to visualize P bodies (green). Nuclei were stained with DAPI (blue). Bar, 10 μm. (**B**) NMuMG cells expressing TWIST were cultured in the presence of rapamycin at the indicated concentrations and in the absence (-) or presence (+) of SBI-0206965 for 24 h. The average number of P-bodies per cell ± SEM (*n* > 150 cells per treatment) was quantified from triplicate experiments. Data were analyzed by one-way ANOVA ^*^P = 0.002. (**C**) Lysates from NMuMG and NMuMG-TWIST cells were examined by Western blotting using antibodies against p62 and GAPDH. (**D**) NMuMG-TWIST cells were treated without (-) or with (+) TGF-β for 24 h. Cells were allowed to recover in fresh media lacking (-) or containing rapamycin (20 nM) (+). Cell lysates were examined by Western blotting using antibodies against p62 and GAPDH.

To further support a role for autophagy in P-body clearance, we also examined NMuMG cells overexpressing TWIST since these cells constitutively contain a large number of P-bodies. We treated TWIST-expressing cells for 24 h with rapamycin, an inhibitor of mTORC1 and a known inducer of autophagy [26]. We observed a dose-dependent decrease in P-body content in rapamycin-treated cells, consistent again with a role for autophagy in P-body clearance (Figure 6A and 6B). Rapamycin induces autophagy by blocking the inhibitory phosphorylation of ULK1 by mTORC1 [27]. ULK1 is a serine/threonine-protein kinase that phosphorylates Beclin-1 to promote autophagy [28]. Therefore, we asked whether the inhibition of ULK1 would counteract the effects of rapamycin. Addition of the ULK1 inhibitor, SBI-0206965, attenuated the ability of rapamycin to promote the clearance of P-bodies in TWIST-overexpressing cells (Figure 6B). These results are consistent with an important role for autophagy in the clearance of P-bodies and suggest that autophagy is compromised in the TWIST-overexpressing cells. Consistent with this suggestion, we found that these cells expressed elevated levels of p62/SQSTM1, an autophagy substrate that accumulates when autophagy is disrupted [29]. This high level of p62 was decreased in cells treated with rapamycin (Figure 6C and 6D). Collectively, our results support an important role for autophagy in the clearance of P-bodies.

### P-body formation is required for induction of EMT by TGF-β

**Figure 7:**
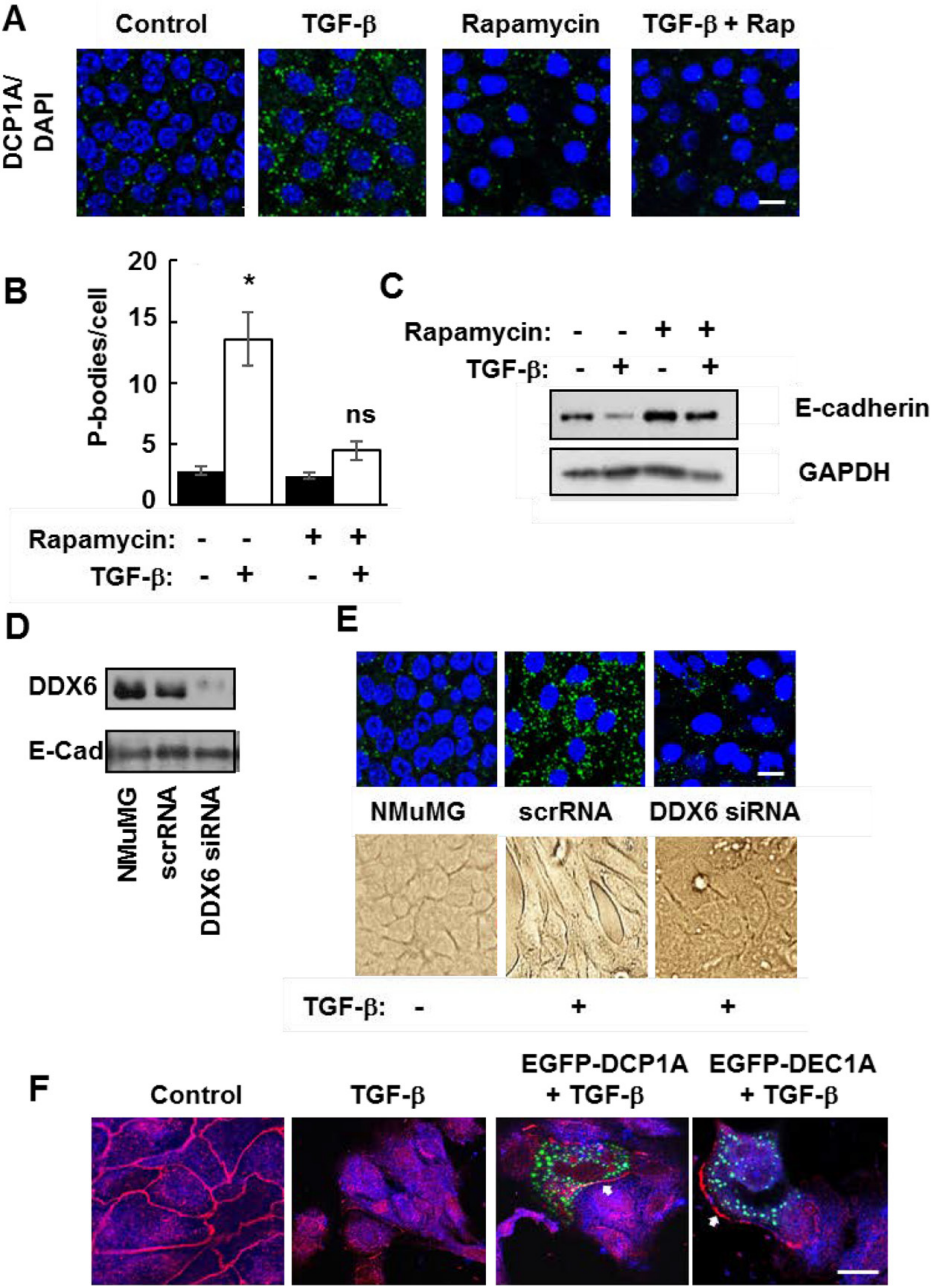
Functional P-bodies are important for EMT (**A**) NMuMG cells were left untreated (Control) or were treated with TGF-β, rapamycin (20 nM), or a combination of TGF-β and rapamycin (TGF-β + Rap) for 24 h. Cells were fixed and stained with antibodies against DCP1A (green). Nuclei were stained with DAPI (blue). Bar, 10 μm. (**B**) Average number of P-bodies per cell ± SEM (n > 150 cells per treatment) from the experiment in panel A was quantified from triplicate experiments. ^*^P < 0.003 compared to control cells. ns, not significant. (**C**) NMuMG cells were treated without (-) or with (+) TGF-β in the absence (-) or presence (+) of rapamycin (20 nM) for 24 h. Cell lysates were examined by Western blotting using antibodies against E-cadherin or GAPDH. (**D**) NMuMG cells were transfected with siRNA against DDX6 (DDX6 siRNA) or a scrambled siRNA (scrRNA) and cultured for 48 h. Transfected cells were compared to untransfected cells (NMuMG) by Western blotting with antibodies against DDX6 and E-cadherin. (**E**) NMuMG cells transfected with siRNA against DDX6 (DDX6 siRNA) or a scrambled siRNA (scrRNA) and cultured for 48 h were then treated without (-) or with (+) TGF-β. Cells were fixed and stained with antibodies against DCP1A (green). Nuclei were stained with DAPI (blue). Bar, 10 μm. Cells also were imaged by light microscopy (lower panel). (**F**) NMuMG cells were transfected with an empty vector or a vector expressing EGFP-DCP1A (green). Control and transfected cells were treated with TGF-β for 24 h and stained with antibodies against E-cadherin (red). Bar, 10 μm. The arrows indicate the location of E-cadherin at the plasma membrane of EGFP-DCP1A-expressing cells.

The correlation between the accumulation of P-bodies and the transition of cells to a mesenchymal phenotype suggested that P-body induction and EMT might be linked. Since rapamycin promoted P-body clearance through autophagy in NMuMG-TWIST cells, we examined its effects on wild type NMuMG cells treated with TGF-β. The accumulation of P-bodies in cells treated with TGF-β was mostly blocked by rapamycin (Figure 7A and 7B). Similarly, rapamycin largely attenuated the TGF-β-stimulated loss of E-cadherin (Figure 7C). To explore further a direct role for P-bodies in EMT, we knocked down the expression of DDX6, a dead-box helicase that acts as a scaffold for their assembly [30, 31]. We transfected NMuMG cells with siRNA directed against the DDX6 mRNA. Cells receiving the DDX6 siRNA, but not a scrambled siRNA, had reduced levels of DDX6 (Figure 7D). These cells failed to form P-bodies in response to TGF-β and failed to undergo the morphological changes consistent with EMT (Figure 7E).

P-bodies are dynamic structures that can form and disassemble in response to modulators of protein synthesis [8]. The ectopic expression of DCP1A forces the assembly of large P-bodies that appear to be nonfunctional as they fail to disassemble in response to emetine or cycloheximide [13]. Therefore, to disrupt P-body function by another mechanism, we transiently overexpressed DCP1A in NMuMG cells and examined its effects on TGF-β-induced EMT. Cells were treated with TGF-β for 48 h, fixed and stained for E-cadherin. In untreated control cells, E-cadherin localized largely to cell-cell junctions as expected (Figure 7F). Control and nontransfected cells exhibited a marked redistribution of E-cadherin away from cell-cell junctions when treated with TGF-β. This change in location of E-cadherin is consistent with cells undergoing EMT [20]. However, in those NMuMG cells that expressed EGFP-DCP1A, E-cadherin was retained at the plasma membrane and cells maintained an epithelial morphology. These studies suggest that the formation of functional P-bodies is an essential process for cells to undergo EMT.

### P-body formation is required for metastasis

**Figure 8:**
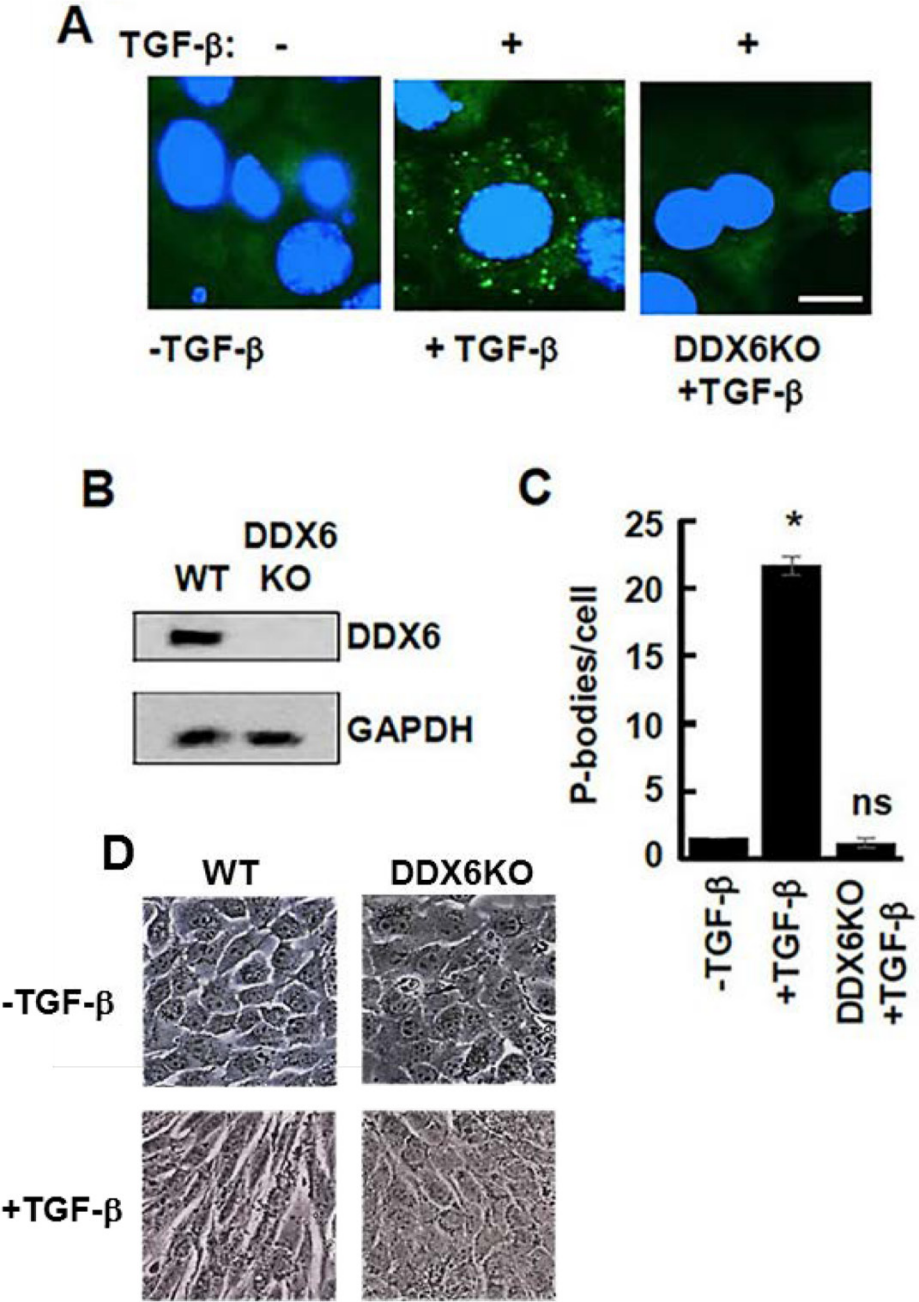
Lack of TGF-β-induced EMT in DDX6-deficient 4T1 cells (**A**) 4T1 mammary carcinoma cells and 4T1 cells lacking DDX6 (DDX6KO) were treated without (-) or with (+) TGF-β for 48 h. Cells were fixed and stained with antibodies against DCP1A (green). Nuclei were stained with DAPI (blue). Bar, 10 μm. (**B**) Lysates from 4T1 cells and 4T1 cells lacking DDX6 (DDX6KO) were analyzed by Western blotting using antibodies against DDX6 and GAPDH. (**C**) Average number of P-bodies per cell ± SEM (*n* > 100 cells per treatment) from the experiment in panel A was quantified from triplicate experiments. ^*^P < 0.001 compared to control, untreated cells. ns, not significant. (**D**) 4T1 cells (WT) or 4T1 cells lacking DDX6 (DDX6KO) were examined by light microscopy before (-TGF-β) and after (+TGF-β) treatment for 48 h with TGF-β.

**Figure 9:**
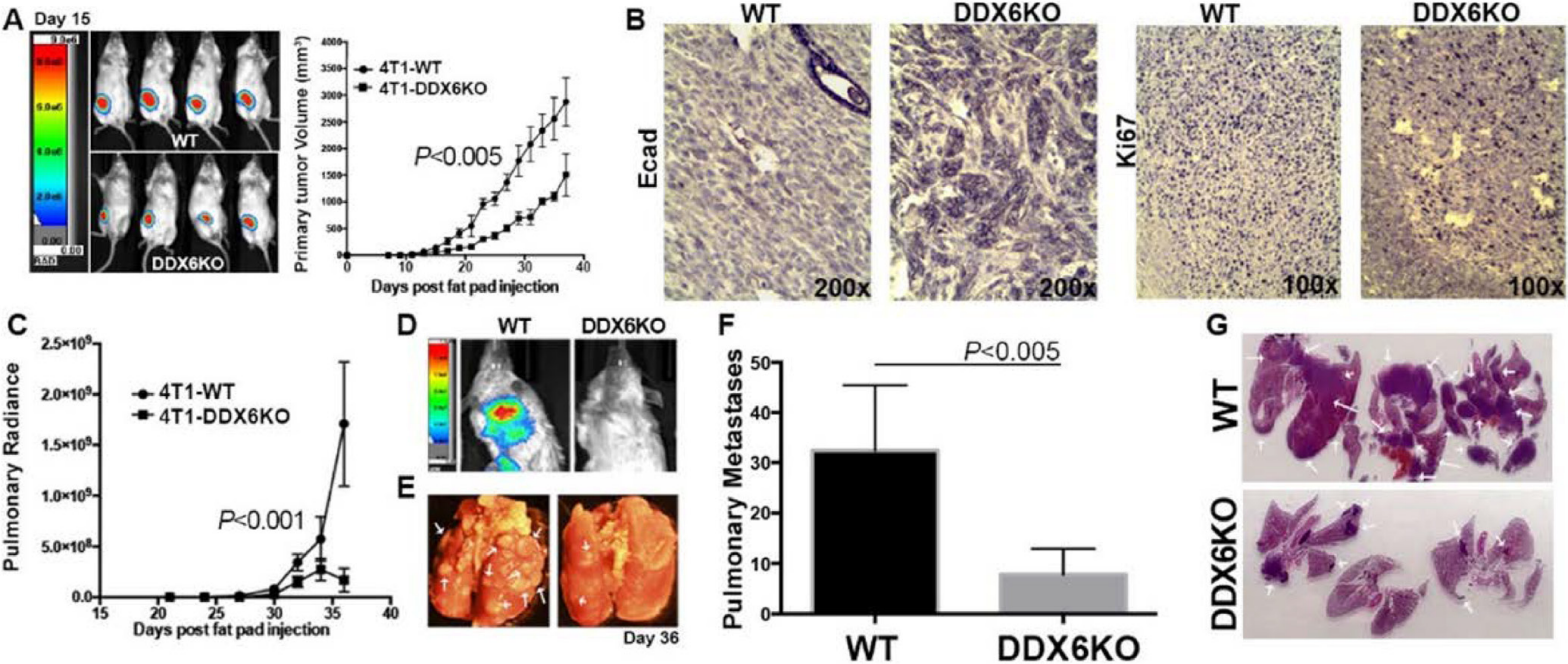
DDX6 is important for metastasis (**A**) 4T1 (WT) and DDX6 deleted 4T1 cells (DDX6KO) expressing firefly luciferase were engrafted onto the mammary fat (2.5x10^4^ cells/mouse). Deletion of DDX6 resulted in reduced primary tumor growth as assessed by bioluminescence and caliper measurements. (**B**) Upon necropsy, the mammary fatpad tumors were removed, fixed and stained via immunohistochemistry for expression of E-cadherin (Ecad) and the proliferive marker Ki67. Images are representative of three separate tumors for each group. (**C**) Pulmonary metastasis was quantified in WT and DDX6K0 tumor-bearing animals by bioluminescence readings taken at the indicated time points. (**D**) Representative bioluminescent images from control and DDX6KO tumor bearing mice. (**E**) Numbers of pulmonary metastases were confirmed upon necropsy and fixation of lung tissues. Arrows indicate pulmonary metastases. (**F**) Quantification of the number of pulmonary metastases in WT and DDX6KO tumor-bearing animals. (**G**) H&E stained histological sections from lungs of three different WT and DDX6KO tumor-bearing animals. Arrows indicate pulmonary metastases. Graphical data in panels (A), (C) and (F) are the mean ± SD of *n* = 4 mice per group resulting in the indicated *P* values.

We then asked if a failure of cells to form P-bodies and undergo EMT would block *in vivo* metastasis. For this experiment, we used our bioluminescent 4T1 model of stage IV breast cancer [32, 33]. *In vitro*, 4T1 cells display an epithelial phenotype, but undergo a robust EMT during primary tumor formation within the mammary fat pad of fully immune-competent Balb/c mice [34]. To block P-body formation, we disrupted the gene for DDX6 by CRISPR gene editing using dCas-Fokl fusion proteins to avoid off-target effects [35]. These cells lacked any detectable DDX6 and failed to accumulate P-bodies or to adopt a mesenchymal morphology when treated with TGF-β (Figure 8A–8D). Upon orthotopic engraftment onto the mammary fat pad, cells deleted for DDX6 demonstrated slightly reduced primary tumor growth as compared to control cells (Figure 9A). Consistent with our *in vitro* data demonstrating the requirement of DDX6 for the induction of EMT, immunohistological staining demonstrated several areas of junctional E-cadherin positivity in DDX6 deleted tumors (Figure 9B). Additionally, staining for the proliferative marker Ki67 appeared to be diminished in DDX6 deleted tumors, consistent with the observed decrease in tumor growth (Figure 9B). Pulmonary metastatic tumor growth was also significantly reduced in DDX6-deleted mammary tumors as determined by bioluminescent imaging (Figure 9C and 9D). Further analysis revealed that not only the size, but the number of pulmonary metastatic nodules was also reduced in the DDX6-deleted tumor bearing mice (Figure 9E–9G). Overall, these data suggest that DDX6 knockout not only inhibits primary and metastatic tumor growth but also decreases systemic dissemination, consistent with inhibition of EMT.

## DISCUSSION

The connection between autophagy and cancer is a complicated one. Autophagy can be tumor suppressive during early stages of tumorigenesis, but tumor promoting at later stages. For example, haploinsufficiency of BECN1 is frequently observed in several forms of human cancer including breast [36–38]. In addition, the positive modifier of autophagy PTEN is tumor suppressive, while the negative effector BCL2 is a tumor promoter [39]. During the later stages of tumor progression, autophagy is thought to be important for tumor cell survival such that autophagy inhibitors like chloroquine or hydroxychloroquine can sensitize certain cells to chemotherapeutic agents [16, 40–42].Similarly, TGF-β also has a paradoxical function in breast cancer where it acts as a tumor suppressor in early stages of disease, but promotes EMT and tumor progression in the metastatic setting [43]. The similarities in the divergent roles for TGF-β and autophagy in cancer progression raises the interesting possibility that these events may be connected by the abilities of each to modulate EMT through the induction and clearance of P-bodies.

During the process of EMT induced by TGF-β, P-bodies accumulate as autophagy is attenuated. Following the removal of TGF-β, these P-bodies largely disappear as autophagy resumes since this clearance of P-bodies is blocked by small molecule inhibitors of autophagy and is enhanced by rapamycin, which stimulates autophagy by enhancing the activity of ULK1. Our observations suggest that this P-body formation is an integral step for EMT as the treatment of NMuMG cells with rapamycin, which prevents P-body accumulation, and the knockout of DDX6, a factor critical for P-body formation, both block TGF-β induced EMT. Thus, factors that negatively affect autophagy and thus promote P-body accumulation might reasonably be expected to promote EMT and tumor cell dissemination. This is consistent with our *in vivo* analyses using the 4T1 model system. As we demonstrated recently, the highly aggressive 4T1 cells clearly undergo EMT during *in vivo* metastasis [34]. The knockout of DDX6 from these cells, which prevents both P-body formation and TGF-β-induced EMT, strongly attenuates the metastatic ability of these cells. Overall, our studies suggest therapeutic targeting of DDX6 and P-body formation as a potential means to inhibit the induction of EMT and therefore block metastasis.

## MATERIALS AND METHODS

### Cells

NMuMG, and 4T1 cells were obtained from American Type Culture Collection (ATCC). Human mammary epithelial (HMLE) cells were generously provided by Dr. Robert Weinberg (The Whitehead Institute, Cambridge, MA). NMuMG cells were cultured in Dulbecco’s modified Eagle’s medium (DMEM) with 4.5 g/l glucose, 10% fetal bovine serum (FBS), 50 units/ml penicillin, 50 μg/ml streptomycin and 20 μg/ml insulin. HMLE cells were cultured in mammary epithelial growth medium (Lonza):F12 media (Mediatech) (1:1) supplemented with 20 μg/ml insulin, 5 ng/ml human epidermal growth factor, 0.25 μg/ml hydrocortisone and 50 units/ml penicillin. NMuMG and HMLE cells transfected to express TWIST were described previously [44]. For the expression of EGFP-DCP1A, we transfected NMuMG cells with pT7-EGFP-C1-HsDCP1A, a gift from Elisa Izaurralde (Addgene plasmid #25030) [45], using Lipofectamine 2000.

### DDX6 knockdown and knockout

To reduce the level of expression of DDX6, NMuMG cells were transfected with a mixture of 4 DDX6 siRNAs (siGENOME SMARTpool siRNA mixture) (Dharmacon) or with a nontargeting siRNA using Lipofectamine RNAiMAX (Invitrogen). Knockdown was verified by Western blotting with antibodies against DDX6. The dimeric CRISPR RNA-guided Fokl nucleases and Csy4-based multiplex gRNA expression system [35] was used to generate the DDX6 knockout cell line. Two annealed target-site oligoduplexes designed by Zifit Targeter [46] and a constant region oligoduplex were assembled with BsmBI-digested pSQT1313 in a single-step ligation. 3 μg of the ligated vector, 1 μg of pSQT1601 expressing Csy4 RNase and RNA-guided Fokl-dCas9 fusion nucleases, and 0.2 μg of pBABE Puro were transfected into luciferase-expressing 4T1 cells. pSQT1313 and pSQT1601 were gifts from Keith Joung (Addgene plasmids #53370 and #53369) [35]. After 48 h, cells were treated with 5 μg/ml puromycin for clonal selection. Genomic DNA and cell lysates from selected colonies were analyzed by the PAGE-genotyping method [47] and Western blot analysis to screen for clones with DDX6 knockout. Disruption of both alleles of DDX6 was confirmed by DNA sequencing.

### P-body formation and clearance

Cells were treated with sodium arsenite (500 µM) for 1h or with TGF-β1 (R&D Systems, 240-B-101) (10 ng/ml) for the indicated times to induce P-bodies. In some experiments, actinomycin D (Sigma-Aldrich, A1410) (5 μg/ml), the TGF-β receptor inhibitor SB-431542 (Tocris Biosciences, 1614) (2 µM), rapamycin (Sigma Aldrich, R8781) (10–40 nM), or rapamycin (20 nM) plus the ULK1 inhibitor SBI-0206965 (Xcess Biosciences, M60268-2s) (5 µM) were added to modulate P-body formation. For P-body clearance assays, cells were washed with phosphate-buffered saline (PBS) and then allowed to recover in fresh media in the absence of TGF-β for the indicated times. In some experiments, one of the autophagy inhibitors N2,N4-bis(phenylmethyl)-2,4-quinazolinediamine (DBeQ) (Sigma-Aldrich, SML0031) (0.625-2.5 µM) or N4-(7-chloro-4-quinolinyl)-N1,N1-dimethyl-1,4-pentanediamine diphosphate salt (chloroquine) (Sigma-Aldrich, C6628) (10 µM) were added during P-body clearance.

For the detection of P-bodies and autophagosomes by immunofluorescence, cells were fixed with 10% ice cold methanol for 10 min, permeabilized with 1% Triton X-100 in PBS, and blocked with PBS containing 10% goat serum, 0.05% Tween 20, and 1 mg/ml BSA. Cells were immunostained using the indicated antibodies against DCP1A, G3BP1, or LC3A/B. Bound primary antibodies were detected using AlexaFluor 488-conjugated goat anti-mouse IgG and/or AlexaFluor 594-conjugated goat anti-rabbit IgG secondary antibodies (Invitrogen). Slides were examined using an EVOS FL imaging system, or a Zeiss LSM 710 confocal microscope. P-bodies were quantified as described [48] using ImageJ to set a threshold mask (Otsu Thresholding Filter), which allowed only P-bodies (puncta) to be analyzed. The pixel range of P-bodies were set at a range between 30 and 270 units. This range was considered to have staining above background. The number of P-bodies and nuclei were counted in an image or field containing at least 25 cells to determine the number of P-bodies per cell. Data are expressed as the mean ± standard error of the mean (SEM) from 3 independent biological replicates. Means were compared by Student’s *t* test for comparisons between two samples or Analysis of Variance (ANOVA) for comparisons of 3 or more samples. Significance was accepted if *P* < 0.05.

### *In vivo* metastasis

Control and DDX6-deleted 4T1 cells engineered to express firefly luciferase were resuspended in PBS (50 μl) and orthotopically engrafted onto the second mammary fat pad of 4 week old Balb/c mice (2.5 × 10^4^ cells/mouse)(Jackson Labs, Bar Harbor, ME). Primary tumor growth and metastasis development were assessed by using digital calipers (Fisher Scientific, Waltham, MA), and via weekly bioluminescent imaging using the Advanced Molecular Imager (AMI) (Spectral Instruments, Tucson, AZ). Tumor volumes were calculated by using the following equation: Tumor Volume = (x^2^) (y) (0.5) where “x” is the tumor width and “y” is the tumor length. Upon necropsy primary tumors and lungs from all animals were removed and fixed in 10% formalin and dehydrated in 70% ethanol for histological sectioning and gross visualization of pulmonary metastatic nodules. All animal studies were performed in accordance with the animal protocol procedures approved by the Institutional Animal Care and Use Committee of Purdue University.

### Antibodies

The following antibodies were used in this study: DCP1A (Santa Cruz, 56-Y), SYK (Santa Cruz, N19), G3BP1 (BD Biosciences, 611126), LC3-A/B (Cell Signaling Technology, D3U4C), p62 (Abcam, ab56416), E-cadherin for immunoblot (Santa Cruz, H10), DDX6 (Sigma Aldrich, P0067), GAPDH (Ambion, AM4300), SMAD2/3 (Cell Signaling Technology, 3102), phospho-SMAD2 (Cell Signaling Technology 3101), E-cadherin for IHC (BD biosciences, 610182), Ki67 (BD biosciences, 550609), AlexaFluor 594-conjugated goat anti-rabbit IgG (Invitrogen), AlexaFluor 594-conjugated goat anti-mouse IgG (Invitrogen), and AlexaFluor 488-conjugated goat anti-mouse IgG (Invitrogen), biotin-conjugated goat anti-mouse IgG (Jackson). For immunohistochemistry biotinylated secondary antibodies were detected using the ABC elite kit in combination with 3-3**′**-diaminobenzidine (Vector).
